# Cry1F Resistance in Fall Armyworm *Spodoptera frugiperda*: Single Gene versus Pyramided Bt Maize

**DOI:** 10.1371/journal.pone.0112958

**Published:** 2014-11-17

**Authors:** Fangneng Huang, Jawwad A. Qureshi, Robert L. Meagher, Dominic D. Reisig, Graham P. Head, David A. Andow, Xinzi Ni, David Kerns, G. David Buntin, Ying Niu, Fei Yang, Vikash Dangal

**Affiliations:** 1 Department of Entomology, Louisiana State University Agricultural Center, Baton Rouge, Louisiana, United States of America; 2 Department of Entomology and Nematology, University of Florida, Institute of Food and Agricultural Sciences, Southwest Florida Research and Education Center, Immokalee, Florida, United States of America; 3 Insect Behavior and Biocontrol Unit, USDA-ARS CMAVE, Gainesville, Florida, United States of America; 4 Department of Entomology, North Carolina State University, Vernon G. James Research and Extension Center, Plymouth, North Carolina, United States of America; 5 Monsanto Company, St. Louis, Missouri, United States of America; 6 Department of Entomology, University of Minnesota, St. Paul, Minnesota, United States of America; 7 USDA, Agricultural Research Service, Crop Genetics and Breeding Research Unit, Tifton, Georgia, United States of America; 8 UGA-Griffin Campus, Department of Entomology, the University of Georgia, Griffin, Georgia, United States of America; Kansas State University, United States of America

## Abstract

Evolution of insect resistance to transgenic crops containing *Bacillus thuringiensis* (Bt) genes is a serious threat to the sustainability of this technology. However, field resistance related to the reduced efficacy of Bt maize has not been documented in any lepidopteran pest in the mainland U.S. after 18 years of intensive Bt maize planting. Here we report compelling evidence of field resistance in the fall armyworm, *Spodoptera frugiperda* (J.E. Smith), to Cry1F maize (TC 3507) in the southeastern region of the U.S. An F_2_ screen showed a surprisingly high (0.293) Cry1F resistance allele frequency in a population collected in 2011 from non-Bt maize in south Florida. Field populations from non-Bt maize in 2012–2013 exhibited 18.8-fold to >85.4-fold resistance to purified Cry1F protein and those collected from unexpectedly damaged Bt maize plants at several locations in Florida and North Carolina had >85.4-fold resistance. In addition, reduced efficacy and control failure of Cry1F maize against natural populations of *S. frugiperda* were documented in field trials using Cry1F-based and pyramided Bt maize products in south Florida. The Cry1F-resistant *S. frugiperda* also showed a low level of cross-resistance to Cry1A.105 and related maize products, but not to Cry2Ab2 or Vip3A. The occurrence of Cry1F resistance in the U.S. mainland populations of *S. frugiperda* likely represents migration of insects from Puerto Rico, indicating the great challenges faced in achieving effective resistance management for long-distance migratory pests like *S. frugiperda*.

## Introduction

Fall armyworm, *Spodoptera frugiperda* (J.E. Smith), is a well-known long-distance migratory insect that is distributed from Argentina to Canada [1]. In the U.S., populations from overwintering areas in south Texas (TX) and south Florida (FL) migrate annually into various regions across the country [2]. *S. frugiperda* is a major target of both Bt maize and Bt cotton in North and South America [3], [4]. In 2013 alone, approximately 50 MHa of Bt crops were planted in the Americas for insect pest management [5], [6]. Effective insect resistance management (IRM) is crucial to ensure the long-term durability of these Bt crops [7]–[9]. Resistance monitoring must be addressed in IRM plans for Bt crops [10]. Although disagreements over the definition of ‘field resistance’ still exist [9], [11]–[13], the possibility of field resistance should be considered when there is a field control failure or significantly reduced efficacy [11]. Based on this criterion, field resistance to Bt crops has been clearly documented in at least four cases around the world [9], [11], including resistance of *S. frugiperda* to Cry1F maize in Puerto Rico [14], [15].

In recent years, unexpected survival of *S. frugiperda* on Cry1F maize has been reported on several occasions in the southeastern U.S. and in Brazil (F.H., R.L.M., J.A.Q., and D.D.R., unpublished data). However, scientific documentation of field resistance to Bt maize in *S. frugiperda* has not been reported anywhere except Puerto Rico [16]. During 2011–2013, an F_2_ screen, diet-incorporated bioassays, greenhouse tests, and field studies with various maize products (Table S1) were conducted in four southeastern U.S. states and the results documented that the unexpected survival of *S. frugiperda* on Cry1F maize in the region was due to resistance. The occurrence of field resistance of *S. frugiperda* in the U.S. mainland indicates a great challenge in resistance management for migratory targets of Bt crops.

## Results and Discussion

In 2011, a total of 142 F_2_ two-parent families of *S. frugiperda* were established using single-pair mating of field-collected individuals, which included 70 families from Rapides and Franklin parishes in central and northeast Louisiana (LA) and 72 families from Collier County in south FL. F_2_ neonates of these families were screened on leaf tissue of Herculex I (HX1) maize expressing the Cry1F protein. The F_2_ screen showed that Cry1F resistance alleles were not rare in the LA and FL populations. The parents of 47.2% families in the two populations were found to carry ≥1 resistance allele (Table 1). For the LA population, parents of 49 families were negative for the presence of resistance alleles (genotype SSSS), 14 families carried 1 resistance allele (RSSS), and 7 families carried 2 resistance alleles (RSRS or RRSS). Among the 72 FL families, only 26 were negative, while 15, 25, 5, and 1 families were identified to carry 1, 2, 3 (RRRS), and 4 (RRRR) resistance alleles, respectively. The Cry1F resistance allele frequency estimated using a multinomial Bayesian statistical model (Methods S1) was 0.103 with a 95% credibility interval (CI) of 0.070 to 0.141 for the LA population and 0.293 with a 95% CI of 0.242 to 0.347 for the FL population. The resistant families initiated with five neonates per plant in the greenhouse grew well and survived on 40–80% of the Cry1F maize plants after 13 d (Table S2). A significant level of resistance (>270-fold) was also observed when these families were tested against purified Cry1F protein in diet-incorporated bioassays (Table 2).

**Table 1:** Expected Cry1F resistance allele frequency (RAF) and corresponding 95% credibility interval (CI) in Louisiana (LA) and Florida (FL) populations of *Spodoptera frugiperda.*

Population	No. F_2_ families screened	No. RAs (genotype) in the two parents of each family					Total no. RAs	RAF(95% CI)
		0 (SSSS)	1 (RSSS)	2 (RSRS or RRSS)	3 (RRRS)	4 (RRRR)		
LA	70	49	14	7	0	0	28	0.103 (0.070, 0.141)
FL	72	26	15	25	5	1	84	0.293 (0.242, 0.347)

R = resistance allele, S = susceptible allele. Number of resistance alleles in the two parents of each family was estimated using the standard monogenic inheritance model by comparing observed and expected survivorship in the F_2_ screen (Tables S7, S9, S10, S11).

**Table 2:** Susceptibility of *Spodoptera frugiperda* collected from multiple locations to purified Cry1F protein using diet-incorporated bioassays.

Insect population	State/county/parish	Year	Host source	LC_50_ (95% CL) (µg/g)	Resistance ratio	% growth inhibition at 10 µg/g (Mean ± SEM)
SS	LA, FL, TX	2008–2013	NBt	0.37 (0.27, 0.49)	-	100±0.0 e
LA-RD-34	Rapides, LA	2011	NBt	>100	>270	28.8±6.1bc
FL-39	Collier, FL	2011	NBt	>100	>270	41.8±4.0 cd
LA-RD-nBt-12	Rapides, LA	2012	NBt	23.1 (17.3, 34.2)	62.4	85.6±1.3 e
LA-FK-nBt-12	Franklin, LA	2012	NBt	10.9 (8.2, 15.3)	29.5	97.2±0.3 e
GA-GB-nBt-12	Tift, GA	2012	NBt	1.33 (1.00, 1.76)	3.6	n/a
GA-VT-nBt-12	Tift, GA	2012	NBt	4.94 (1.50, 75.6)	13.4	60.4±7.9 d
FL-CL-nBt-12	Collier, FL	2012	NBt	6.97 (4.12, 14.4)	18.8	87.1±3.0 e
FL-HD-nBt-12	Hendry, FL	2012	NBt	>31.6 (0.0±0.0)	>85.4	31.4±4.9 c
FL-MI-nBt-13	Miami-Dade, FL	2013	NBt	7.35 (5.35, 10.8)	19.9	86.9±4.1e
FL-HD-nBt-13	Hendry, FL	2013	NBt	29.5 (18.9, 55.8)	79.7	33.3±4.3c
FL-CL-nBt-13	Collier, FL	2013	NBt	20.7 (13.4, 41.0)	55.9	18.3±4.7abc
FL-AC-12	Alachua. FL	2012	Bt	>31.6 (0.0±0.0)	>85.4	−1.5±7.4a
FL-CL-Bt-12	Collier, FL	2012	Bt	>31.6 (0.0±0.0)	>85.4	27.9±4.3bc
FL-CL-Bt-13	Collier, FL	2013	Bt	>31.6 (0.0±1.9)	>85.4	0.5±5.5a
NC-Bt-13	Hyde, NC	2013	Bt	>31.6 (7.1±5.1)	>85.4	5.9±6.0ab

Larval mortality was calculated as the number of dead larvae divided by the total number of larvae in the test. During this study, three Cry1F-susceptibile (SS) strains (SS-FL, SS-LA, and SS-TX) were used as references. SS-FL was initiated from larvae collected from Hendry Co., FL in 2011; SS-LA was established from insects collected from Franklin Parish, LA in 2008; and SS-TX was developed from insects collected from Hidalgo Co., TX in 2013. All three SS strains were highly susceptible to both Cry1F maize plants and Cry1F protein in diet. Because the overall performance on maize plants and diet were similar among the three strains, SS was used to denote all three strains unless mentioned specifically. FL-HD-nBt-12 was collected from a heavily infested non-Bt sweet corn field that was close to an early-planted Bt maize field. The Bt maize field was heavily damaged by *S. frugiperda* and the population infesting the non-Bt sweet corn was believed to be the progeny of moths that came out of the Bt maize field. LA-RD-24 and FL-39 were two resistant families isolated from populations from Rapides Parish, LA and Collier Co., FL, respectively, using the F_2_ screen. FL-CL-nBt-12, FL-CL-nBt-13, FL-CL-Bt-12, and FL-CL-Bt-13 were collected from non-Bt and Bt plants in two field trials in Collier Co., FL in 2012 and 2013. LA: Louisiana, FL: Florida, GA: Georgia, NC: North Carolina. NBt: non-Bt maize, Bt: Bt maize. The LC_50_ value of a population was considered to be greater than the highest Cry1F concentration tested if its larval mortality was <50% at the highest concentration in the bioassays. Limited by the cost of Cry1F protein, the highest concentrations used in the bioassays varied depending on the sources of the populations. The highest concentration assayed for LA-RD-24 and FL-39 was 100 µg/g, while it was 31.6 µg/g for other populations. Mortality at 100 µg/g was 20.6±3.9% for LA-RD-24 and 0.0±0.0% for FL-39. Mortality at 31.6 µg/g was 7.1±5.1% for FL-SC-Bt-13 and zero for FL-HD-nBt-12 and all other populations collected from Bt maize plants. Resistance ratio was calculated as the LC_50_ of the field populations divided by that of the SS strain. Analysis of variance for growth inhibition: F_14,46_ = 59.75, *P*<0.0001. Mean values for growth inhibition followed by a common letter were not significantly different at α = 0.05 (Tukey’s HSD test). n/a: Data are not available.

We interpreted the high Cry1F resistance allele frequency estimated by the F_2_ screen in the FL population as an indication of field resistance as defined above. To confirm this hypothesis, 13 additional populations of *S. frugiperda* were collected from LA, Georgia (GA), FL, and North Carolina (NC) during 2012–2013 (Table 2), which included 9 populations (2 LA, 2 GA, 5 FL) from non-Bt maize and 4 populations from Cry1F maize. Two of the four populations from Cry1F maize were collected from fields that showed unexpected damage by feral populations of *S. frugiperda*, which included one from FL and one from NC. The other two of the four populations from Cry1F maize were collected from two field trials in FL in 2012 and 2013. Diet-incorporated bioassays showed that, relative to the Cry1F-susceptible (SS) strains, larvae of *S. frugiperda* collected from non-Bt maize were 3.6- to >85.4-fold less susceptible to purified Cry1F protein (Table 2). All four populations collected from Cry1F plants in FL and NC were highly resistant (>85.4-fold) to Cry1F protein. No significant mortality was observed at the Cry1F concentration of 31.6 µg/g, the highest concentration tested, for any of the four populations. The results confirmed that the unexpected damage by *S. frugiperda* observed in the fields in FL and NC was due to resistance to the Cry1F protein in the plants.

**Figure 1:**
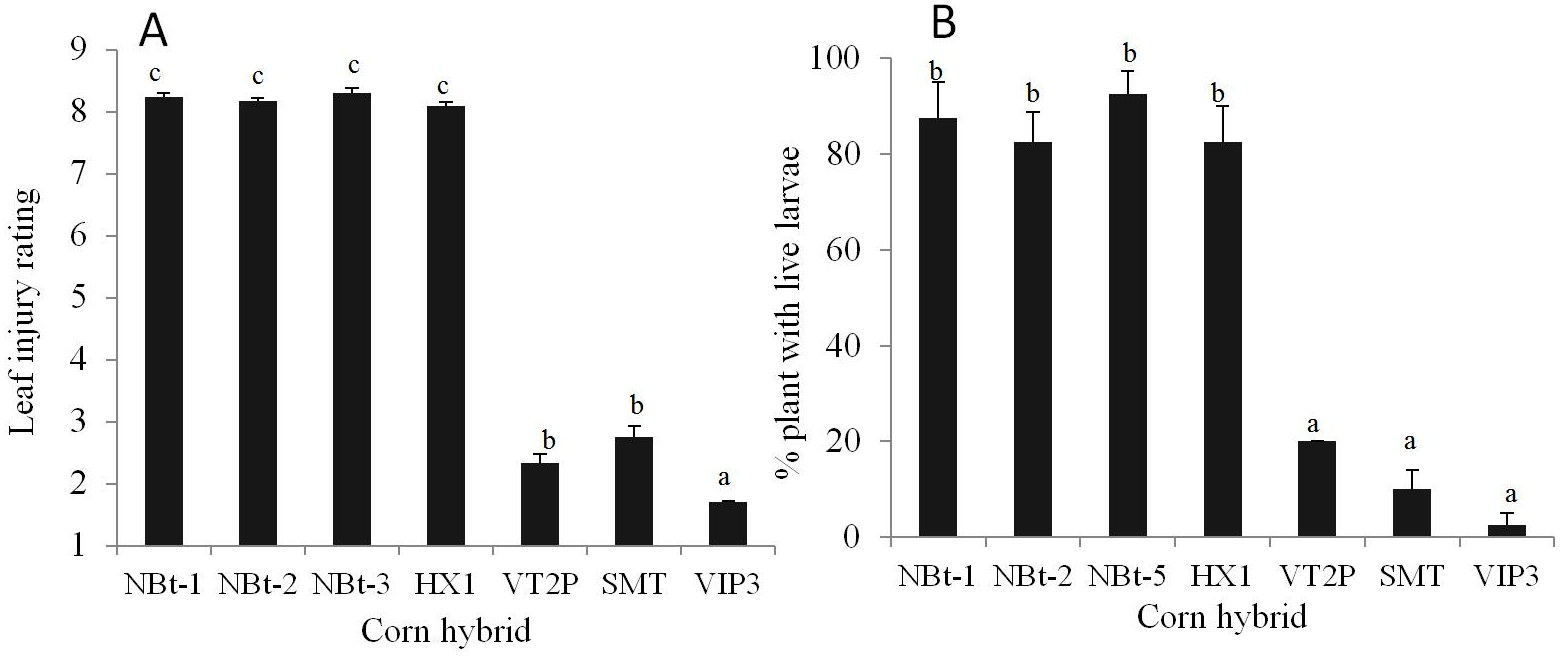
Leaf injury ratings (A) and occurrence of feral populations of *Spodoptera frugiperda* (B) on non-Bt and Bt maize containing single and pyramided genes in a field trial in 2013 in Collier Co., Florida. Leaf injury ratings were measured at V9–V12 plant stages using Davis’s 1–9 scale^17^, while larval occurrence was recorded at R1 plant stage. NBt-1, Pioneer 31P40; NBt-2, DKC 61-22; NBt-5, N78N-GT; HX1, Pioneer 31D59; VT2P, DKC 61-49; SMT, DKC 62-08; VIP3, N78N-3111. Bars represent means (±SEM) and those with a common letter were not significantly different at α = 0.05 (Tukey’s HSD test).

There also was clear evidence of Cry1F resistance in the field when trials were conducted in 2012 and 2013 at the location in FL where *S. frugiperda* were collected for the F_2_ screen. In 2012, an average leaf injury rating of 4.3 (Davis 1–9 scale) [17] due to the damage by *S. frugiperda* was recorded on Cry1F maize plants during the V2–V10 plant stages [18] (Table S3). Additional greenhouse tests showed that five out of 20 Cry1F plants each infested with 10 F_1_ neonates of *S. frugiperda* collected from non-Bt plants in the FL field trial were heavily injured, with a leaf injury rating of 6–9, and five live 4^th^–5^th^ instars were recovered from the five plants (1 larva/plant) after 12 d (Table S4). In contrast, the Cry1F plants killed all of the SS larvae placed on them and had virtually no leaf injury. More importantly, the field trial in 2013 showed that Cry1F plants were essentially ineffective against the feral populations of *S. frugiperda* (Fig. 1). There were no significant differences in the leaf injury ratings and the percentage of plants containing live larvae of *S. frugiperda* between the non-Bt and Cry1F maize (HX1) plants. Both non-Bt and Cry1F plants were heavily injured by *S. frugiperda*, with a leaf injury rating of 8.24 on the non-Bt maize and 8.09 on HX1 at V9–V12 and >80% plants at the R1 plant stage contained large, live larvae (most of which were 5^th^ instars) (Fig. 1). Diet-incorporated bioassays showed that the larvae collected from the non-Bt maize plants had 18.8-fold (for FL-CL-NBt-2012) and 55.9-fold (FL-CL-NBt-2013) resistance to Cry1F protein (Table 2). As described above, for the two populations (FL-CL-Bt-2012 and FL-CL-Bt-2013) collected from the Cry1F plants, no mortality was observed at 31.6 µg/g of diet. Thus, the performance of the Cry1F maize in the 2012 trial showed reduced efficacy of Cry1F because the non-Bt maize plants had significantly greater leaf injury, while the 2013 trial demonstrated failure of Cry1F against *S. frugiperda*. The results of the field trials confirmed that field resistance to Cry1F maize in *S. frugiperda* had occurred in FL and NC.

The geographical range and distribution of Cry1F resistance in *S. frugiperda* in the mainland U.S. remains unknown. A recent independent study found an resistance allele frequency of 0.132 to Cry1F in three samples of *S. frugiperda* collected from Palm Beach and Hendry counties in FL in 2011 and 2012 [16]. They found no unexpected field survival, but one population collected from Palm Beach in 2012 showed a resistance allele frequency of 0.247. Although unexpected field survival of *S. frugiperda* has not been reported in LA, the resistance allele frequency (0.103) of the LA populations detected in this study was also relatively high. The results of our study, together with other published data, indicate that the range of Cry1F resistance in *S. frugiperda* may be geographically extensive in the southeast coastal region of the U.S.

The factors that led to the field resistance of *S. frugiperda* to Cry1F maize in FL and NC are unknown. Local selection pressure due to the planting of Bt maize appears not to be a major factor driving the development of field resistance. In most years, *S. frugiperda* in the U.S. mainland overwinters only in south FL and south TX [2], [19]. Maize is not a major crop in FL, which had a total planting area of <40,000 ha/year [5]. A high proportion of maize in the state is sweet corn, and most sweet corn does not contain Bt genes. In addition, *S. frugiperda* is a polyphagous insect with a wide host range [19]. For these reasons, local selection pressure by Bt maize in FL should be limited. Although it is unclear if local selection caused by the use of Bt microbial insecticides is a contributing factor, the more plausible reason for the field resistance appears to be the migration of resistant populations from Puerto Rico through other Caribbean islands to FL. Northerly movement of FL populations along the U.S. East Coast has been documented for years [20]. This hypothesis is supported by a recent comparative study of mitochondrial haplotype ratios in *S. frugiperda* [21]. The study showed that the Puerto Rico populations of *S. frugiperda* had only very limited interactions with TX populations, but could have substantial exchanges with FL populations. In addition, the areas with unexpected damage by *S. frugiperda* on Bt maize also match the expected migration patterns of *S. frugiperda* from the Caribbean islands to the mainland U.S. that were generated based on weather patterns [20].

While further studies are warranted to reveal the geographical ranges and factors leading to field resistance in the U.S. mainland, effective management of Cry1F- resistant populations of *S. frugiperda* is needed to ensure the continued success of Bt crop technologies. To generate essential information for IRM, additional F_2_ screen, and laboratory, greenhouse, and field studies were performed to analyze the cross-resistance to other commonly used Bt proteins and Bt maize products containing single and pyramided traits.

**Figure 2:**
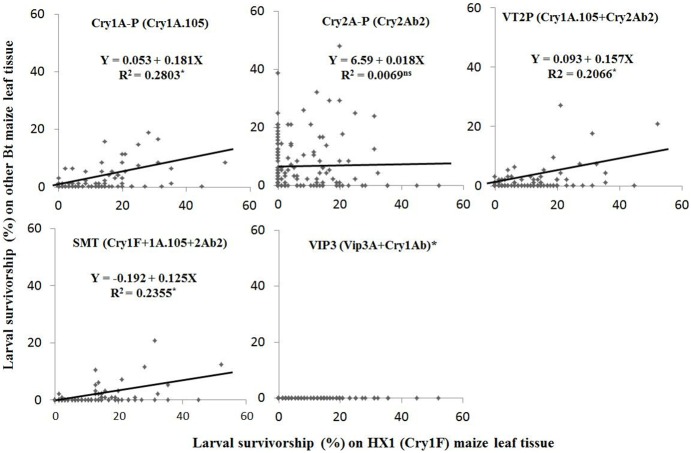
Correlation analysis on larval survivorship (%) of 142 F_2_ two-parent families between HX1 vs. five other Bt maize products. Analysis was performed by treating survival on HX1 as the independent variable (X) and survival on the other five Bt products as the dependent variable (Y) [38]. HX1, Pioneer 31D59; Cry1A-P, an experimental line expressing the Cry1A.105 protein; Cry2A-P, an experimental line expressing the Cry2Ab2 protein; VT2P, DKC 61-49; SMT, DKC 62-08; VIP3, N78N-3111. “*” indicates statistical significance (*P*<0.05), while “ns” indicates not significant (*P*≥0.05).

We analyzed the cross-resistance of *S. frugiperda* between HX1 and five other Bt maize products based on the survival of the 142 families in an F_2_ screen that was performed simultaneously with the F_2_ screen against HX1 mentioned above. The five Bt maize products included two experimental Bt maize lines, Cry1A.105Ln (Cry1A-P) and Cry2Ab2Ln (Cry2A-P), as well as three commercial products: Genuity VT Double (VT2P), Genuity SmartStax (SMT), and Agrisure Viptera 3111 (VIP3). Cry1A-P and Cry2A-P produce a single Bt protein, Cry1A.105 and Cry2Ab2, respectively, whereas VT2P expresses both Cry1A.105 and Cry2Ab2 (Table S1) [22]. SMT produces six Bt proteins including the two in VT2P and Cry1F for controlling Lepidoptera plus Cry3Bb1 and Cry34/35Ab1 for Coleoptera. VIP3 produces three Bt proteins including Vip3A and Cry1Ab for Lepidoptera and mCry3A for Coleoptera. Correlation analysis showed that there was a significantly (*P*<0.05) positive relationship in larval survival of the 142 families between Cry1F maize and three other maize products, namely, Cry1A-P, VT2P, and SMT, but not with Cry2A-P or VIP3 (Fig. 2). The correlation coefficients calculated based on larval survivorship between HX1 and other products were 0.534 (*P*<0.05) for Cry1A-P, 0.461 (*P*<0.05) for VT2P, and 0.491 (*P*<0.05) for SMT, but only 0.021 (not significant) for Cry2A-P (Table S5). No correlation coefficients could be calculated with VIP3 which killed all of the F_2_ larvae in the 142 families and has previously been reported to be extremely toxic towards *S. frugiperda* [23]–[25]. The results suggest that some level of cross-resistance exists between HX1 and Cry1A-P, VT2P, and SMT, but not VIP3 and Cry2A-P.

To understand the cross-resistance patterns observed in the F_2_ screen, diet-incorporated bioassays were conducted to determine the susceptibility of a known Bt-susceptible strain collected from TX in 2013 (SS-TX) and a resistant strain (FL-39) to five individual purified Cry proteins: Cry1Aa, Cry1Ab, Cry1Ac, Cry1A.105, and Cry2Ab2. FL-39 was isolated from a two-parent family of the FL population using the F_2_ screen mentioned above. Relative to SS-TX, FL-39 exhibited 4.8-fold less susceptibility to Cry1A.105, while susceptibility to Cry2Ab2 was similar between SS-TX and FL-39 (Table 3). Individual Cry1Aa, Cry1Ab, and Cry1Ac proteins were not very effective against either strain. LC_50_s of the three proteins were ≥23.8 µg/g against the two strains, and larvae of both strains showed a considerable weight gain at 31.6 µg/g. Survival of SS-TX and FL-39 was also evaluated in the greenhouse on whole plants of YieldGard corn borer (YG), VIP3, and three Bt maize lines: Cry1A-P, Cry2A-P, and Cry2Ab2Hn (Cry2A-HP, an experimental line expressing a ‘high level’ of Cry2Ab2 protein). In these tests, YG was virtually ineffective against *S. frugiperda*, with an average of 58.3% plants containing live larvae at 12 d after infestation with five neonates of SS-TX or FL-39 per plant (Table 4). In contrast, no larvae of either strain survived on VIP3. Cry1A-P also killed all of the SS-TX larvae, while 37.5% of the Cry1A-P plants infested with FL-39 contained live larvae. SS-TX and FL-39 survived on 18.8% and 31.3% of the Cry2A-P plants, respectively, but no survivors of either strain were observed on Cry2A-HP. The results of the greenhouse tests further confirmed that some level of cross-resistance exists in *S. frugiperda* between Cry1F and Cry1A.105, but not between Cry1F and Cry2Ab2 or Vip3A.

**Table 3:** Median lethal concentrations (LC_50_) and 95% confidence limits (CL) based on larval mortality of Cry1F-susceptible (SS-TX) and -resistant (FL-39) strains of *Spodoptera frugiperda* to five individual Cry proteins.

Cry protein	Insectstrain	*N*	Slope ± SE	LC_50_ (95% CL)(µg/g)	Resistanceratio	% growth inhibition at31.6 µg/g (mean ± SEM)
Cry1Aa	SS-TX	495	-	>31.6		72.6±4.4 bc
	FL-39	512	-	>31.6	-	42.0±10.8 a
Cry1Ab	SS-TX	938	0.83±0.17	28.3 (12.3, 141.8)		62.3±9.3 ab
	FL-39	507	3.20±1.0	23.8 (14.5, 56.7)	−1.2	66.7±4.3 ab
Cry1Ac	SS-TX	608	n/a	>31.6		74.6±7.4 bcd
	FL-39	577	0.83±0.24	29.2 (12.7, 347.8)	−1.1	71.6±2.5 ab
Cry1A.105	SS-TX	576	1.94±0.31	9.0 (6.3, 13.8)		100±0.0 e
	FL-39	640	1.30±0.24	43.5 (27.6, 84.1)	4.8	81.4±4.7 bcd
Cry2Ab2	SS-TX	543	0.87±0.28	17.7 (7.4, 260.4)		94.2±1.9 cde
	FL-39	576	1.28±0.33	12.5 (5.8, 88.6)	−1.4	95.0±1.4 de

SS-TX was developed from insects collected from Hidalgo Co., TX in 2013 and documented to be susceptible to Cry1F maize and Cry1F protein. FL-39 was a resistant family isolated from an FL population collected in 2011 using an F_2_ screen. *n* = total number of neonates assayed. Limited by the amount of Cry proteins available, the highest concentrations used in some bioassays didn’t cause a 50% or greater larval mortality. LC_50_ value of an insect strain was considered to be greater than the highest Cry concentration assayed if its larval mortality was <50% at the highest concentration. Mortality at 31.6 µg/g was 6.7±1.1% for SS-TX and 26.1±9.5% for FL-39 for Cry1Aa, and 24.1±10.2% for SS-TX for Cry1Ac. Resistance ratios for a Cry protein were calculated by dividing the greater LC_50_ value by the smaller one. A negative sign was given if the LC_50_ of FL-39 was smaller than that of SS-TX. Analysis of variance for growth inhibition: F_4,29_ = 22.19, *P*<0.0001 for protein; F_1,29_ = 12.18, *P* = 0.0016 for insect strain; and F_4,29_ = 4.79, *P* = 0.0043 for the interaction. Mean values followed by a common letter in a column were not significantly different at α = 0.05 (Tukey’s HSD test).

**Table 4:** Leaf injury rating and % plants containing live larvae (mean ± SEM) of Cry1F-susceptible (SS-TX) and -resistant (FL-39) strains of *Spodoptera frugiperda* on whole plants of non-Bt and Bt maize containing single or pyramided genes.

Maize hybrid/line	Maize trait	Leaf injury rating after 7 d		% plants containing live larvae after 12 d	
		SS-TX	FL-39	SS-TX	FL-39
Non-Bt	NBt	3.75±0.16 c	4.1±0.15 c	87.5±5.59 e	84.4±5.98 de
Pioneer 31D50	HX1	1.13±0.07 a	4.31±0.33 c	0.00±0.00 a	75.00±10.21 cde
DKC 69–70	YG	3.21±0.36 c	3.50±0.40 c	60.4±6.25 bcde	56.25±6.25 bcd
Cry1A.105Ln	Cry1A-P	1.06±0.06 a	3.31±0.19 c	0.00±0.00 a	37.50±7.22 bc
Cry2Ab2Ln	Cry2A-P	2.06±0.12 b	1.88±0.07 b	18.75±6.25 ab	31.25±6.25 abc
Cry2Ab2Hn	Cry2A-HP	1.00±0.00 a	1.00±0.00 a	0.00±0.00 a	0.00±0.00 a
DKC 64-04	VT2P	1.06±0.06 a	1.19±0.12 a	0.00±0.00 a	0.00±0.00 a
DKC 62-08	SMT	1.00±0.00 a	1.00±0.00 a	0.00±0.00 a	0.00±0.00 a
N78N-3111	VIP3	1.00±0.00 a	1.00±0.00 a	0.00±0.00 a	0.00±0.00 a
Analysis of variance	Insect	*F*_1,75_ = 89.73, *P*<0.0001		*F*_1,75_ = 14.78, *P* = 0.0003	
	Maize	*F*_8,75_ = 196.09, *P*<0.0001		*F*_8,75_ = 66.72, *P*<0.0001	
	Interaction	*F*_8,75_ = 31.6, *P*<0.0001		*F*_8,75_ = 6.39, *P*<0.0001	

Data were pooled for three non-Bt maize hybrids/lines which included DKC 61-22 (NBt-2), N78N-GT (NBt-5), and ExpH (NBt-6). n/a: Information is not available. Mean values followed by a common letter within a parameter measured were not significantly different at α = 0.05 (Tukey’s HSD test).

The observed cross-resistance in the F_2_ screen between HX1 and the two pyramided products VT2P and SMT is likely due to the similar (Cry1A.105) and/or shared (Cry1F in SMT) protein domains in the products. VT2P and SMT contain the same Cry1A.105 gene [22]. Cry1A.105 is a chimeric protein incorporating domains I and II from Cry1Ab or Cry1Ac, domain III from Cry1F, and the C-terminal domain from Cry1Ac [26]. Limited by the technology available, the expression levels of Cry1F or Cry1A.105 in the pyramided Bt maize plants were not determined. Based on the gene structures, the overall amino acid sequence identity of Cry1A.105 to Cry1Ac, Cry1Ab, and Cry1F is 93.6%, 90.0%, and 76.7%, respectively [26]. As shown in both the diet-incorporated bioassays and the whole-plant tests, both Cry1Ab and Cry1Ac were ineffective against *S. frugiperda*. Thus, if *S. frugiperda* develops resistance to Cry1F protein, Cry2Ab2 is the only protein in VT2P and SMT still fully active against *S. frugiperda* (with incomplete resistance to Cry1A.105). Because Cry2Ab2 has a mode of action distinct from that of Cry1F or Cry1A [27], cross-resistance between Cry2Ab2 and Cry1F or Cry1A is unlikely [16], [28]–[31]. The results of our study are consistent with those of a recent protein binding study [32] which showed that *S. frugiperda* shares binding sites for Cry1A.105 and Cry1F. The high effectiveness of VIP3 against *S. frugiperda* is most likely due to the Vip3A protein. As mentioned above, neither YG plants nor purified Cry1Ab protein are very effective against *S. frugiperda*, indicating a limited activity of Cry1Ab protein in VIP3 for the insect. Cross-resistance between Cry1F and Vip3A is unlikely because the two proteins do not share binding sites [33] and was not seen in the case of the Puerto Rico Cry1F-resistant population of *S. frugiperda* [16].

In North and South America, pyramided Bt maize products are becoming prevalent and thus it is necessary to know the performance of these products in order to effectively manage Cry1F-resistant populations of *S. frugiperda*. In a greenhouse trial, we observed no larval survival of either SS-TX or FL-39 on three pyramided Bt products (VT2P, SMT, and VIP3) (Table 4). Niu et al. [25] also showed that these pyramided Bt maize products were effective in controlling a Puerto Rico Cry1F-resistant population of *S. frugiperda* in the greenhouse. To validate the performance of these products in the field, the HX1 field trial in Collier Co., FL in 2013 was extended to include VT2P, SMT, and VIP3 along with closely related non-Bt maize hybrids. As described above, the feral population of *S. frugiperda* at the trial site (FL-CL-nBt-13 and FL-CL-Bt-13) was highly resistant to both HX1 maize (Fig. 1) and purified Cry1F protein (Table 2). The field trial showed that the natural population of *S. frugiperda* caused very limited leaf injury on the pyramided (VT2P, SMT, and VIP3) Bt-plants (1.7–2.8 on the Davis scale), with 2.5–20.0% of the plants containing live larvae (Fig. 1). Some larvae could have moved between plots, but sampling was avoided at the plot ends where this risk was high. A positive correlation was observed between the survival in the F_2_ screen and the open field trial for the three pyramided products, suggesting that the low level of cross-resistance to Cry1A.105 could allow limited survival of Cry1F-resistant *S. frugiperda* on maize plants with pyramided traits related to Cry1A.105.

Our documentation of field resistance of *S. frugiperda* to Cry1F maize in the continental U.S. indicates that the Cry1F-based crop technologies may face a great challenge due to the migration of the Cry1F-resistant populations of *S. frugiperda*. It appears that geographic isolation and withdrawal of Cry1F maize (TC1507) from Puerto Rico [14] were not enough to stop the spread of resistance. Cry1F maize was first registered in 2001 in the U.S. and later commercially planted in Puerto Rico in 2003 for controlling lepidopteran pests including *S. frugiperda*, which is the most important maize pest in the territory [14], [34]. With the extensive use of TC1507 maize products along with several other factors [11], [25], field resistance to Cry1F maize occurred in Puerto Rico in 2006 [14], [15]. Upon an initial confirmation of field resistance in 2006 and as a part of IRM, the commercial sale of Cry1F maize seeds was stopped in Puerto Rico [14], [35]. However, resistance is still persistent after several years of not planting TC1507 products [15], [25], [36]. In addition, unlike Bt resistance in most other insects, the Cry1F resistance in *S. frugiperda* is likely complete resistance [25] and not associated with any fitness costs [16], [37]. Thus, the Cry1F based Bt maize and Bt cotton products currently planted in North and South America could be at risk. For example, Cry1F-resistant *S. frugiperda* could migrate north and damage Bt maize fields. The resistance observed in NC in this study may be a good example of such a situation. In the southern US, resistant populations of *S. frugiperda* could impact WideStrike cotton that contains the Cry1F protein. In Brazil and Argentina, >18 MHa of Bt crops were planted in 2013, much of it targeted against *S. frugiperda* [6]. Therefore, effective IRM for *S. frugiperda* and other similar migratory polyphagous pests will require careful consideration of their movement patterns and of possible Bt crop deployment strategies.

## Materials and Methods

### Insect collections

Third to fifth instars of *S. frugiperda* were collected during 2011–2013 from multiple locations in four southeastern U.S. states: LA, GA, FL, and NC. Insects collected in 2011 were used to establish two-parent families for an F_2_ screen [39]. A total of >1,200 larvae of *S. frugiperda* were collected from sorghum fields in Franklin and Rapides parishes in LA and from non-Bt sweet corn fields in Collier Co. in south FL. Field-collected larvae were reared individually on a meridic diet as described in Yang et al [40]. Newly emerged virgin male and female adults derived from the field collections were paired. Progeny (F_1_) produced by each pair were separately reared on diet and the F_1_ adults were sib-mated within each two-parent family to produce F_2_ offspring. The number of viable F_1_ pupae in each family ranged from 55 to 80 with an average of 76.5±1.0 (mean ± SE) for the LA populations and 50 to 80 with an average of 67.9±1.7 (mean ± SE) for the FL population. The F_2_ neonates were used in an F_2_ screen on Bt maize leaf tissue as described below.

In addition, 13 field populations of *S. frugiperda* were collected during 2012–2013 from Bt and non-Bt maize fields in 10 locations in LA, GA, FL, and NC (Table 2). Sample size was 35 larvae for one population (FL-CL-Bt-13) and 92–300 for other populations. Field-collected larvae were reared on a meridic diet [36] and F_1_ progeny of the field-collected populations, except NC-13, were used to determine the susceptibility to purified Cry1F protein. For NC-13, F_3_ progeny were used in the bioassay. Purified (99.9%) Cry 1F protein was obtained from Case Western Reserve University, Cleveland, Ohio, USA [41].

### F_2_ screen

A total of 142 two-parent families of *S. frugiperda* were established from the field collections in 2011, which included 70 families from LA and 72 from FL (Table 1). Among the 70 LA families, 47 families were collected from Rapides Parish and 23 were from Franklin Parish. F_2_ neonates of the families were screened on leaf tissues of HX1, Cry1A-P, Cry2A-P, VT2P, SMT, and VIP3 maize as described in Yang et al [40]. Limited by the technology available, expression levels of Bt proteins in plants were not measured, but Cry protein expression for a maize hybrid/line was confirmed using the ELISA-based assays (EnviroLogix, Quantiplate kits, Portland, ME. In each family, 96 neonates were placed in 24 wells (4 neonates/well) (Bio-Smart-32, C–D International, Pitman, NJ) containing leaf tissue excised from greenhouse-grown maize plants at V4–V9. The decision to use four neonates per well was based on a previous study to minimize larval cannibalism [40]. All bioassay trays containing maize leaf tissue and larvae of *S. frugiperda* were incubated in environmental chambers maintained at 28°C, ∼50% RH and a 16-h: 8-h (L:D) photoperiod. Fresh leaf tissue was added every 2–3 d. Larval survival and development were recorded after 7 d. Live larvae were separated into two groups based on their growth: small (1^st^ or 2^nd^ instars) and large (≥3^rd^ instars).

### Definition of potential positive families possessing resistance alleles to Cry1F maize

During the study, three SS strains (SS-FL, SS-LA, and SS-TX) were used as references for laboratory bioassays and greenhouse tests. SS-FL was initiated from larvae collected from non-Bt maize fields in Hendry Co., FL in 2011 [36]; SS-LA was established from cotton and maize fields in 2008 in LA [42]; and SS-TX was developed from insects collected from non-Bt maize in TX in 2013. All three SS strains were highly susceptible to both Cry1F maize plants and Cry1F protein in diet. Because the overall performance on maize plants and diet were similar among the three strains, SS was used to denote all three strains unless mentioned specifically. Baseline survival assays showed that all three Cry1F-susceptible strains (SS) of *S. frugiperda* survived well on non-Bt maize leaf tissue after 7 d with an average survivorship of 63.4% and a larval mass of 44.2 mg/larva (Table S6). In contrast, on HX1 leaf tissue, only a small percentage (2.3%) of larvae survived and all survivors were 1^st^ or 2^nd^ instars. The results suggested that survivorship of large larvae (≥3^rd^ instars) in the F_2_ screen on HX1 leaf tissue could be used to identify potential positive families carrying resistance alleles to Cry1F. Correspondingly, ≤2^nd^ instars that survived the F_2_ screen were treated as dead larvae in determining resistance alleles.

Theoretically, if one of the two parents of a family contains a recessive resistance allele, 6.25% of the F_2_ larvae are expected to be homozygous (RR) for Bt resistance and should survive in the F_2_ screen [39]. Based on the baseline survival data of SS, an average of 3.59 [ = 96 (neonates screened)×6.25% (RR frequency)×59.9% (baseline survivorship on HX1)] live larvae were expected in a family in the F_2_ screen if one parent of the family possessed a resistance allele. A χ^2^-test showed that a survival of one larva in a family was not significantly (*P*>0.05) different from the expected survivorship (3.59 larvae/family), and thus a family with one or more survivors was considered as a potential positive family for resistance alleles to Cry1F maize.

### Cry1F resistance confirmation

Based on the larval survival in the F_2_ screen, 21 of the 70 LA families of *S. frugiperda* and 46 of the 72 FL families were identified to be potential positive families (Table S7). To confirm if a potential positive family actually possessed resistance alleles, six strains were established from the survivors of six potential positive families including three families (LA-RD-24, LA-RD-34, and LA-RD-37) from Rapides Parish, LA and three families (FL-13, FL-37, and FL-39) from FL (Table S8). To increase the chance of success in the strain establishments, all F_2_ survivors (both large and small larvae) of a family were transferred to the diet [36] and reared in varied temperatures to synchronize their development. Progeny of the strains established were then selected on Cry1F maize leaf tissue for 1–2 times using the similar methods as described in the F_2_ screen. Initial confirmation for the six potential positive families was performed by measuring larval survival of the potential positive families and SS on HX1 leaf tissue using the same method as described in the F_2_ screen. Then, resistance of three potential positive families (LA-RD-24, LA-RD-34, and FL-39) was reconfirmed on whole plants of greenhouse-grown HX1 plants (Table S2). In the reconfirmation tests, five neonates of a potential positive family were placed into the whorl of a plant at the V6–VT stages. Leaf injury ratings, larval survival, and larval mass were recorded 12–14 d after the initial insect infestation. In addition, non-Bt maize and SS-FL were also included in the tests as the controls of the experiment. A potential positive family was considered to actually possess resistance alleles if it showed a significant survivorship with live ≥3^rd^ instars on the leaf tissue and on whole plants in the confirmation tests.

In addition, susceptibility to purified Cry1F protein of two families (LA-RD-34 and FL-39) that were already confirmed to be resistant to Cry1F maize was examined, along with SS, using a diet-incorporated bioassay [36] (Table 2). In the bioassay, larval survival (both small and large larvae) and masses of live larvae were recorded 7 d after neonate infestations. Corrected dose/mortality data [43] of SS were subjected to probit analysis [38], [44] to determine LC_50_ and 95% CL. For the two resistant families, the LC_50_ value was considered to be greater than the highest Cry concentration (100 µg/g) tested because the larval mortalities were <50% at 100 µg/g. Resistance ratios were calculated using the LC_50_ value of a HX1-resistant strain divided by the LC_50_ of SS. In addition, the percentage of larval growth inhibition at 10 µg/g was calculated as described in Huang et al [45]. Growth inhibition data were analyzed using a one-way analysis of variance (ANOVA) [38]. Comparison among insect strains was made using the Tukey’s HSD test at α = 0.05.

### Estimate of Cry1F resistance allele frequency

Results of the resistance confirmation studies showed that all six potential positive families examined possessed resistance alleles against HX1 maize plants. Diet-incorporated bioassays further confirmed that the survival of *S. frugiperda* on HX1 maize plants was due to resistance to the Cry1F protein in the plants. Therefore, all of the 21 LA and 46 FL potential positive families identified in the F_2_ screen were considered to carry resistance alleles. Revisiting the F_2_ screen data (Table S7), we found that the survivorship of F_2_ progeny of some families in the F_2_ screen was much greater than the expected survival of 3.59 larvae/family, suggesting that there was >1 resistance allele in the two parents of some families. To accurately estimate the resistance allele frequency, a χ^2^-test with the assumption of single-gene Mendelian inheritance was used to determine the number of resistance alleles in the two parents of each family (Tables S7, S9, S10, S11). We then framed a Bayesian statistical model [39], [46], [47] as a multinomial problem to calculate the expected resistance allele frequency and the corresponding 95% CI (Methods S1).

### Susceptibility of field populations of *S. frugiperda* to Cry1F protein

The surprisingly high Cry1F resistance allele frequency in the populations of *S. frugiperda* detected in the F_2_ screen, especially for the FL population, suggests that it should be possible to detect resistance using a convenient dose-response bioassay method [48]. During 2012–2013, a total of 13 field populations of *S. frugiperda* were collected from LA, GA, FL, and NC (Table 2). Susceptibility of these field populations, along with SS, to purified Cry1F protein was determined using the diet-incorporated bioassay method as described above. Limited by the cost of Cry1F protein, these populations were assayed with Cry1F concentrations up to only 31.6 µg/g. LC_50_s and larval growth inhibition (%) were analyzed using the methods mentioned above.

### Survival and leaf injury of natural populations of *S. frugiperda* on non-Bt and HX1 under field and greenhouse conditions

Larval survival and plant injury of natural populations of *S. frugiperda* were evaluated in 2012 and 2013 in the same field (26° 28′N, 81° 26′W) at the Southwest Florida Research and Education Center, University of Florida in Collier Co., FL where insects were collected for the F_2_ screen. The field trials were permitted by the Southwest Florida Research and Education Center, University of Florida. The field work did not involve any endangered or protected species. No human participants, specimens or tissue samples, or vertebrate animals, embryos or tissues were involved in the study. A randomized completely block (RCB) design with four replications was used in both years. There were 200 plants/replication in 2012 and 504 plants/replication in 2013. Only an HX1 hybrid and a closely related non-Bt maize hybrid were included in the trial in 2012, while the test in 2013 also contained three pyramided Bt maize traits (VT2P, SMT, and VIP3) along with closely related non-Bt maize hybrids (Fig. 1). Leaf injury by *S. frugiperda* was rated using Davis’ 1–9 scale [17] in V2–V10 plant stage for the trial in 2012 and V9–V12 plant stages for the trial in 2013. In addition, in the 2013 trial, larval occurrence of *S. frugiperda* was recorded at the R1 plant stage, when the plants showed maximum leaf injury. Occurrence of *S. frugiperda* was not recorded for the trial in 2012. Transformed data [25] on leaf injury ratings and percentage plants containing live larvae were analyzed using a one-way ANOVA [38]. Treatment means for each trial were separated using Tukey’s HSD test at α = 0.05.

In addition, susceptibility to Cry1F protein of field-collected populations (F_1_) from non-Bt and Bt maize plants of the two trials was determined using the diet-incorporated bioassay as described above. Larval survival and plant injury of the field population (F_1_ of FL-CL-nBt-12) collected from non-Bt maize plants in the trial in 2012 along with SS were also tested on whole plants of greenhouse-grown HX1 and non-Bt maize plants to demonstrate the biological activity of HX1 against susceptible *S. frugiperda* and resistance in the field-collected population. In the greenhouse tests, 10 neonates of FL-CL-nBt-12 and SS were placed into the whorl of a plant at V5–V7. Larval survival and leaf injury ratings were recorded at 12 d after insect infestation. A RCB was used in the test with four replications and 5 plants/replication. Transformed data [25] were analyzed using a two-way ANOVA (38). Treatment means were separated using Tukey’s HSD test at α = 0.05.

### Determination of cross-resistance

Cross-resistance of Cry1F-resistant *S. frugiperda* was examined using two methods. First, correlation and regression analyses [38] were performed to examine if there were significant relationships in the survivorship of the 142 families of *S. frugiperda* in the F_2_ screen on leaf tissue of HX1 and the five other Bt maize products. A significant positive correlation would suggest the existence of cross-resistance among Bt maize products. Second, susceptibility of FL-39 and SS-TX to five common individual Cry proteins (Cry1Aa, Cry1Ab, Cry1Ac, Cry1A.105, and Cry2Ab2) was determined using the diet-incorporated bioassay method as described above. All five proteins were provided by Monsanto Company (St. Louis, MO, USA).

### Larval survival and plant injury of SS and Cry1F-resistant *S. frugiperda* on Bt maize plants containing single and pyramided traits

Both greenhouse and field studies were utilized to evaluate if pyramided Bt maize and other related traits were effective against the Cry1F- resistant *S. frugiperda*. In the greenhouse study, larval survival and plant injury of FL-39 and SS-TX were investigated on four non-Bt and eight Bt maize products using a method similar to that described in Niu et al [25]. The eight Bt maize products included five commercial products (HX1, YG, VT2P, SMT, and VIP3) and three experimental lines (Cry1A-P, Cry2A-P, and Cry2A-HP). Expression/non-expression of Bt proteins for a maize hybrid/line was also confirmed using the ELISA-based assays mentioned above. The four non-Bt maize products were genetically closely related to 1–2 of the Bt products. At the V5–V7 plant stages, five neonates of a *S. frugiperda* strain were placed into the whorl of a plant. Leaf injury was rated with Davis’ 1–9 scale at 7 d after larval release, and larval survival was recorded after 12 d. A RCB was used in the tests containing four replications with four plants/replication. Transformed data [25] were analyzed using a two-way ANOVA [38]. Treatment means were separated using Tukey’s HSD test at α = 0.05.

## Supporting Information

Table S1 **Non-Bt and Bt maize products evaluated in this study.**
(DOCX)

Table S2 **Plant injury and larval survival of a Cry1F-susceptible (SS-FL) strain and three Cry1F-resistant families (LA-RD-24, LA-RD-34, and FL-39) of** ***Spodoptera frugiperda*** **on whole plants of non-Bt and Cry1F maize hybrids in the greenhouse.**
(DOCX)

Table S3 **Leaf injury ratings (mean ± SEM) of non-Bt and HX1 plants caused by feral populations of** ***Spodoptera frugiperda*** **in a field trial in Collier Co., FL in 2012.**
(DOCX)

Table S4 **Leaf injury rating and survival (mean ± SEM) of Cry1F-susceptible strain (SS-FL) and a field population (FL-CL-NBt-2012) of** ***Spodoptera frugiperda*** **collected from non-Bt maize from a field in Collier Co., FL in 2012 and tested in the greenhouse.**
(DOCX)

Table S5 **Correlation coefficients of larval survivorship of 142 two-parent families of** ***Spodoptera frugiperda*** **in an F_2_ screen with maize leaf tissue containing single or pyramided Bt genes.**
(DOCX)

Table S6 **Baseline survivorship (mean ± SEM) of a susceptible strain (SS-FL) of** ***Spodoptera frugiperda*** **on leaf tissue of Bt and non-Bt maize plants.**
(DOCX)

Table S7 **Potential positive families (PPF) possessing resistance alleles (RAs) to Cry1F maize that were identified in the F_2_ screen in three populations of** ***Spodoptera frugiperda*** **collected from Louisiana (LA) and Florida (FL).**
(DOCX)

Table S8 **Larval survivorship (%) of potential positive families of** ***Spodoptera frugiperda*** **on leaf tissue of Cry1F maize plants.**
(DOCX)

Table S9 **Baseline survival (mean ± SEM) of Cry1F-susceptible (SS-FL), -resistant (RR), and -heterozygous (RS) genotypes of** ***Spodoptera frugiperda*** **on leaf tissue of HX1 and non-Bt (NBt) maize plants.**
(DOCX)

Table S10 **Expected frequency of genotypes of in the F_2_ progeny of a two-parent family of** ***Spodoptera frugiperda.***
(DOCX)

Table S11 **Six different parental (P_1_) crosses of two-parent families and the related frequencies and genetic models.**
(DOCX)

Methods S1 **Supplementary methods.**
(DOCX)
