# Supplementary material for: Cry1F Resistance in Fall Armyworm Spodoptera frugiperda: Single Gene versus Pyramided Bt Maize
Source: PLoS One. 2014 Nov 17;9(11):e112958. doi: 10.1371/journal.pone.0112958 (PMC4234506; doi:10.1371/journal.pone.0112958)
Supplement: Table S1 — Non-Bt and Bt maize products evaluated in this study. (DOCX) [file pone.0112958.s001.docx]

**Table S1**. Non-Bt and Bt maize products evaluated in this study.

| Maize product | Bt traits | Abbreviation | Event | Bt gene |
| --- | --- | --- | --- | --- |
|  |  |  |  |  |
| Pioneer 31P40 | Non-Bt | NBt-1 | Closely related to Pioneer 31D59 | |
| DKC 61-22 | Non-Bt | NBt-2 | Closely related to DKC 61-49 and DKC 61-21 | |
| DKC 63-45 | Non-Bt | NBt-3 | Closely related to DKC 61-49 and DKC 63-87 | |
| DKC 67-86 | Non-Bt | NBt-4 | Closely related to DKC 69-70 | |
| N78N-GT | Non Bt | NBt-5 | Closely related to N78N-3111 | |
| ExpL | Non-Bt experimental line | NBt-6 | Closely related to Cry1A.105Ln and Cry2Ab2Ln | |
| EepH | Non-Bt experimental line | NBt-7 | Closely related to Cry2Ab2Hn | |
| Pioneer 31D59 | Herculex I | HX1 | TC1507 | Cry1F |
| Cry1A.105Ln | Experimental line | Cry1A-P | n/a | Cry1A.105 |
| Cry2Ab2Ln | Experimental line with low expression of Cry2Ab2 protein | Cry2A-P | n/a | Cry2Ab2 |
| Cry2Ab2Hn | Experimental line with high expression of Cry2Ab2 protein | Cry2A-HP | n/a | Cry2Ab2 |
| DKC 69-70 | YieldGard | YG | MON810 | Cry1Ab |
| DKC 61-49 | Genuity VT Double Pro | VT2P | MON89034 | Cry1A.105, Cry2Ab2 |
| DKC 63-87 |  |  |  |  |
| DKC 61-21 | Genuity SmartStax | SMT | MON89034+ TC1507 + MON88017+DAS-59112-7 | Cry1A.105, Cry2Ab2, Cry1F, Cry3Bb1, Cry34/35Ab |
| DKC 62-08 |  |  |  |  |
| N78N-3111 | Agrisure Viptera 3111 | VIP3 | Bt11+MIR162+MIR604 | Vip3A, Cry1Ab, mCry3A |

The same abbreviations for maize products were used throughout the text, tables, and figures. n/a: not available. Expression/non-expression of Bt proteins for a maize hybrid/line was confirmed using ELISA-based assays (EnviroLogix, Quantiplate kits, Portland, ME).
