# Supplementary material for: Cry1F Resistance in Fall Armyworm Spodoptera frugiperda: Single Gene versus Pyramided Bt Maize
Source: PLoS One. 2014 Nov 17;9(11):e112958. doi: 10.1371/journal.pone.0112958 (PMC4234506; doi:10.1371/journal.pone.0112958)
Supplement: Table S2 — Plant injury and larval survival of a Cry1F-susceptible (SS-FL) strain and three Cry1F-resistant families (LA-RD-24, LA-RD-34, and FL-39) of Spodoptera frugiperda on whole plants of non-Bt and Cry1F maize hybrids in the greenhouse. (DOCX) [file pone.0112958.s002.docx]

**Table S2.** Plant injury and larval survival of a Cry1F-susceptible (SS-FL) strain and three Cry1F-resistant families (LA-RD-24, LA-RD-34, and FL-39) of *Spodoptera frugiperda* on whole plants of non-Bt and Cry1F maize hybrids in the greenhouse.

| Insect | Maize plant | No. plants | Plant stages | Days after neonate release | Leaf injury rating (range) | Larval survival | | |
| --- | --- | --- | --- | --- | --- | --- | --- | --- |
|  |  |  |  |  |  | % plants with live larvae | Total no. live larvae | Larval mass, mg/larva (range) |
| SS-FL | NBt-1 | 10 | V8–V9 | 12 | 8.2 (7–9) | 70% | 15 | 120.4 (39–230) |
| Combined resistant families | NBt-1 | 18 | V8–V9 | 13–14 | 7.3 (6–9) | 100 | 27 | 130.0 (41–273) |
| SS-FL | HX1 | 14 | V6–V7 | 14 | 1.2 (1–2) | 0 | 0 | --- |
| LA-RD-24 | HX1 | 10 | VT | 13 | 3.6 (1–6) | 80 | 12 | 47.4 (16–123) |
| LA-RD-34 | HX1 | 10 | VT | 13 | 2.5 (1–4) | 40 | 5 | 136.2 (9–323) |
| FL-39 | HX1 | 10 | V9–V10 | 13 | 8.9 (8–9) | 80 | 14 | 233.8 (50–419) |

Five neonates were released in the whorl of each plant. LA-ED-24, LA-RD-34, and FL-39 were three Cry1F-resistant families isolated from field populations collected from Louisiana (LA-RA-24 and LA-RD-34) and Florida (FL-39) using F_2_ screen. SS-FL was collected from non-Bt maize in Hendry Co., FL in 2011 and documented to be susceptible to Cry1F. “Combined resistant families” includes LA-RD-24 and FL-39. Survival of LA-RD-34 on non-Bt maize plants was not evaluated. LA-RD-24 and LD-RD-34 were two resistant families isolated from the Rapides population in LA, while FL-39 was established from the FL population. Leaf injury was rated using the Davis 1–9 scale^17^.
