# Supplementary material for: Cry1F Resistance in Fall Armyworm Spodoptera frugiperda: Single Gene versus Pyramided Bt Maize
Source: PLoS One. 2014 Nov 17;9(11):e112958. doi: 10.1371/journal.pone.0112958 (PMC4234506; doi:10.1371/journal.pone.0112958)
Supplement: Table S3 — Leaf injury ratings (mean ± SEM) of non-Bt and HX1 plants caused by feral populations of Spodoptera frugiperda in a field trial in Collier Co., FL in 2012. (DOCX) [file pone.0112958.s003.docx]

**Table S3.** Leaf injury ratings (mean ± SEM) of non-Bt and HX1 plants caused by feral populations of *Spodoptera frugiperda* in a field trial in Collier Co., FL in 2012.

| Maize | Plant stages | | |
| --- | --- | --- | --- |
|  | V2–V4 | V5–V7 | V8–V10 |
| NBt-1 | 5.5 ± 0.6 a | 7.1 ± 0.3 b | 7.4 ± 0.1 b |
| HX1 | 4.5 ± 0.2 a | 4.1 ± 0.1 a | 4.4 ± 0.1 a |
| Analysis of variance | *F*_1,3_ = 9.55  *P* = 0.0537 | *F*_1,3_ = 107.85  *P* = 0.0019 | *F*_1,3_ = 133.55  *P* = 0.0014 |

A randomized complete block design was used with four replications. Data were recorded from 25 randomly selected plants/replication. Leaf injury was rated using the Davis 1–9 scale (17). Mean values followed by a common letter in a column were not significantly different at α = 0.05 (Tukey's HSD test).
