# Supplementary material for: Cry1F Resistance in Fall Armyworm Spodoptera frugiperda: Single Gene versus Pyramided Bt Maize
Source: PLoS One. 2014 Nov 17;9(11):e112958. doi: 10.1371/journal.pone.0112958 (PMC4234506; doi:10.1371/journal.pone.0112958)
Supplement: Table S4 — Leaf injury rating and survival (mean ± SEM) of Cry1F-susceptible strain (SS-FL) and a field population (FL-CL-NBt-2012) of Spodoptera frugiperda collected from non-Bt maize from a field in Collier Co., FL in 2012 and tested in the greenhouse. (DOCX) [file pone.0112958.s004.docx]

**Table S4.** Leaf injury rating and survival (mean ± SEM) of Cry1F-susceptible strain (SS-FL) and a field population (FL-CL-NBt-2012) of *Spodoptera frugiperda* collected from non-Bt maize from a field in Collier Co., FL in 2012 and tested in the greenhouse.

| Insect | Maize | Leaf injury rating | % plants containing live larvae |
| --- | --- | --- | --- |
| SS-FL | NBt-1 | 8.1 ± 0.4 b | 100 ± 0.0 c |
|  | HX1 | 1.2 ± 0.1 a | 0.0 ± 0.0 a |
| FL-CL-NBt-2012 | NBt-1 | 8.7 ± 0.3 b | 100 ± 0.0 c |
|  | HX1 | 2.5 ± 0.6 a | 20.8 ± 8.0 b |
| Analysis of variance | Insect | F_1,8_ = 5.92, *P* =0.0409 | F_1,8_ = 7.39, *P* = 0.0263 |
|  | Maize | F_1,8_ = 134.92, *P* < 0.0001 | F_1,8_ = 295.06, *P* < 0.0001 |
|  | Interaction | F_1,8_ =2.42, *P* = 0.158 | F_1,8_ = 5.94, *P* = 0.0408 |

SS-FL was collected from Hendry Co., FL in 2011 and documented to be susceptible to Cry1F. Plants were infested with 10 neonates/plant at V5–V7 stages. Davis 1–9 scale (17). Among the 20 Cry1F plants that were infested with FL-CL-NBt-2012, 5 plants contained live larvae (1 larva/plant) with a larval development stage of 4^th^–5^th^ instar and a leaf injury rating of 6–9 at 12 d after infestations. Mean values followed by a common letter in a column were not significantly different at α = 0.05 (Tukey's HSD test).
