# Supplementary material for: Cry1F Resistance in Fall Armyworm Spodoptera frugiperda: Single Gene versus Pyramided Bt Maize
Source: PLoS One. 2014 Nov 17;9(11):e112958. doi: 10.1371/journal.pone.0112958 (PMC4234506; doi:10.1371/journal.pone.0112958)
Supplement: Table S5 — Correlation coefficients of larval survivorship of 142 two-parent families of Spodoptera frugiperda in an F2 screen with maize leaf tissue containing single or pyramided Bt genes. (DOCX) [file pone.0112958.s005.docx]

**Table S5**. Correlation coefficients of larval survivorship of 142 two-parent families of *Spodoptera frugiperda* in an F_2_ screen with maize leaf tissue containing single or pyramided Bt genes.

|  | HX1 | Cry1A-P | Cry2A-P | VT2P | SMT | VIP3 |
| --- | --- | --- | --- | --- | --- | --- |
| HX1 |  | 0.534* | 0.021 | 0.461* | 0.491* | n/a |
| Cry1A-P | 0.519* |  | 0.132 | 0.460* | 0.600* | n/a |
| Cry2A-P | -0.053 | 0.099 |  | 0.064 | 0.058 | n/a |
| VT2P | 0.450* | 0.45* | 0.035 |  | 0.613* | n/a |
| SMT | 0.483* | 0.594* | 0.032 | 0.609* |  | n/a |
| VIP3 | n/a | n/a | n/a | n/a | n/a |  |

Data used for the correlation analysis were based on 7-d survivorship of 142 F_2_ two-parent families of *S. frugiperda* in an F_2_ screen. Correlation coefficients above the diagonal line were calculated based on the larval survivorships observed for all F_2_ families that were screened, while values below the diagonal line were based on only the families that survived at least one of the corresponding two Bt maize products in the F_2_ screen. Correlation coefficient is significant (*P* < 0.05). n/a: correlation analysis was not available.
