# Supplementary material for: Cry1F Resistance in Fall Armyworm Spodoptera frugiperda: Single Gene versus Pyramided Bt Maize
Source: PLoS One. 2014 Nov 17;9(11):e112958. doi: 10.1371/journal.pone.0112958 (PMC4234506; doi:10.1371/journal.pone.0112958)
Supplement: Table S6 — Baseline survivorship (mean ± SEM) of a susceptible strain (SS-FL) of Spodoptera frugiperda on leaf tissue of Bt and non-Bt maize plants. (DOCX) [file pone.0112958.s006.docx]

**Table S6**. Baseline survivorship (mean ± SEM) of a susceptible strain (SS-FL) of *Spodoptera frugiperda* on leaf tissue of Bt and non-Bt maize plants.

| Maize product | Bt traits | Abbreviation | Event | Bt gene | Larval survival after 7 d (%) | | | Larval mass (mg/larva) |
| --- | --- | --- | --- | --- | --- | --- | --- | --- |
|  |  |  |  |  | ≤2^nd^ instar | ≥3^rd^ instar | Total |  |
| Non-Bt | Non-Bt maize | NBt | --- | --- | 3.5 ± 0.9 | 59.9 ± 2.2 | 63.4 ± 2.3 d | 44.2 ± 2.3 c |
| Pioneer 31D59 | Herculex I | HX1 | TC1507 | Cry1F | 2.3 ± 1.5 | 0.0 ± 0.0 | 2.3 ± 1.5 a | 3.5 ± 0.8 ab |
| Cry1A.105Ln | Experimental line | Cry1A-P | n/a | Cry1A.105 | 0.4 ± 0.4 | 0.0 ± 0.0 | 0.4 ± 0.4 a | 2.1 ab |
| Cry2Ab2Ln | Experimental line with a low expression of Cry2Ab2 protein | Cry2A-P | n/a | Cry2Ab2 | 9.8 ± 3.2 | 0.8 ± 0.5 | 10.5 ± 3.2 b | 4.6 ± 1.6 ab |
| Cry2Ab2Hn | Experimental line with a high expression of Cry2Ab2 protein | Cry2A-HP | n/a | Cry2Ab2 | 0.8 ± 0.8 | 0.0 ± 0.0 | 0.8 ± 0.8 a | 3.4 ab |
| DKC 69-70 | YieldGard | YG | MON810 | Cry1Ab | 28.1 ± 2.2 | 3.1 ± 1.3 | 31.3 ± 3.4 c | 7.0 ± 1.0 b |
| DKC 63-87 | Genuity VT Double Pro | VT2P | MON89034 | Cry1A.105, Cry2Ab2 | 1.6 ± 0.9 | 0.0 ± 0.0 | 1.6 ± 1.6 a | 1.5 ± 0.5 a |
| DKC 61-21 | Genuity SmartStax | SMT | MON89034+ TC1507 + MON88017+DAS-59112-7 | Cry1A.105, Cry2Ab2, Cry1F, Cry3Bb1, Cry34/35Ab | 0.0 ± 0.0 | 0.0 ± 0.0 | 0.0 ± 0.0 a | --- |
| N78N-3111 | Agrisure Viptera 3111 | VIP3 | Bt11+MIR162+MIR604 | Vip3A, Cry1Ab, mCry3A | 0.0 ± 0.0 | 0.0 ± 0.0 | 0.0 ± 0.0 a | --- |

SS-FL was initiated from larvae collected from non-Bt maize fields in Hendry County in Florida in 2011. Non-Bt maize products evaluated included Pioneer 31G66 (NBt-1), DKC 61-22 (NBt-2), DKC 63-45 (NBt-3), DKC 67-86 (NBt-4), N78N-GT (NBt-5), and ExpL (NBt-6) (see Table S1). Baseline survival of *S. frugiperda* was determined using the same method as described in the F_2_ screen. Survivors on leaf tissue after 7 d were separated into two groups: (1) small larvae (≤2^nd^ instars) with a body mass of ≤5 mg/larva and (2) large larvae (≥3^rd^ instars) with a body mass of >5 mg/larva. There were at least four replications for each maize product with 32 larvae/replication. ANOVA: *F*_8,59_ = 113.42, *P* < 0.0001 for larval survival and *F*_6,36_ = 60.13, *P* < 0.0001 for larval mass. Mean values followed by the same letter in a column were not significantly different at α = 0.05 (Tukey's HSD test).
