# Supplementary material for: Cry1F Resistance in Fall Armyworm Spodoptera frugiperda: Single Gene versus Pyramided Bt Maize
Source: PLoS One. 2014 Nov 17;9(11):e112958. doi: 10.1371/journal.pone.0112958 (PMC4234506; doi:10.1371/journal.pone.0112958)
Supplement: Table S7 — Potential positive families (PPF) possessing resistance alleles (RAs) to Cry1F maize that were identified in the F2 screen in three populations of Spodoptera frugiperda collected from Louisiana (LA) and Florida (FL). (DOCX) [file pone.0112958.s007.docx]

**Table S7.** Potential positive families (PPF) possessing resistance alleles (RAs) to Cry1F maize that were identified in the F_2_ screen in three populations of *Spodoptera frugiperda* collected from Louisiana (LA) and Florida (FL).

| Family | No. live larvae in the family | No. RAs in the two parents of the family |
| --- | --- | --- |
| Rapides Parish, LA | | |
| LA-RD-1 | 5 | 1 |
| LA-RD-2 | 8 | 2 |
| LA-RD-13 | 3 | 1 |
| LA-RD-18 | 1 | 1 |
| LA-RD-20 | 7 | 1 |
| LA-RD-21 | 14 | 2 |
| LA-RD-23 | 18 | 2 |
| LA-RD-26 | 8 | 2 |
| LA-RD-27 | 14 | 2 |
| LA-RD-32 | 1 | 1 |
| LA-RD-34 | 5 | 1 |
| LA-RD-37 | 10 | 2 |
| LA-RD-38 | 3 | 1 |
| LA-RD-39 | 2 | 1 |
| LA-RD-41 | 4 | 1 |
| LA-RD-47 | 2 | 1 |
| LA-RD-50 | 4 | 1 |
| LA-RD-56 | 2 | 1 |
| LA-RD-59 | 2 | 1 |
| Franklin Parish, LA | | |
| LA-RD-3 | 8 | 2 |
| LA-RD-25 | 4 | 1 |
|  |  |  |
|  |  |  |
| Collier County, FL | | |
| FL-1 | 2 | 1 |
| FL-4 | 2 | 1 |
| FL-10 | 1 | 1 |
| FL-12 | 5 | 1 |
| FL-13 | 31 | 3 |
| FL-15 | 1 | 1 |
| FL-17 | 16 | 2 |
| FL-18 | 14 | 2 |
| FL-19 | 5 | 1 |
| FL-20 | 2 | 1 |
| FL-21 | 15 | 2 |
| FL-23 | 6 | 1 |
| FL-25 | 9 | 2 |
| FL-27 | 2 | 1 |
| FL-29 | 1 | 1 |
| FL-30 | 20 | 2 |
| FL-31 | 19 | 2 |
| FL-32 | 25 | 3 |
| FL-34 | 12 | 2 |
| FL-35 | 1 | 1 |
| FL-37 | 23 | 3 |
| FL-39 | 33 | 3 |
| FL-41 | 13 | 2 |
| FL-42 | 8 | 2 |
| FL-44 | 16 | 2 |
| FL-45 | 22 | 2 |
| FL-46 | 10 | 2 |
| FL-48 | 2 | 1 |
| FL-52 | 14 | 2 |
| FL-53 | 14 | 2 |
| FL-54 | 2 | 1 |
| FL-55 | 10 | 2 |
| FL-58 | 15 | 2 |
| FL-59 | 12 | 2 |
| FL-61 | 8 | 2 |
| FL-62 | 15 | 2 |
| FL-63 | 32 | 3 |
| FL-65 | 6 | 1 |
| FL-66 | 11 | 2 |
| FL-67 | 47 | 4 |
| FL-68 | 1 | 1 |
| FL-69 | 19 | 2 |
| FL-70 | 12 | 2 |
| FL-72 | 19 | 2 |
| FL-73 | 13 | 2 |
| FL-74 | 18 | 2 |

Based on the baseline survival (Table S6), a family with one or more survivors was considered as a PPF possessing RAs to Cry1F maize. To determine the number of RAs in the two parents of a PPF, baseline survivorship of known Cry1F-susceptible (SS-FL) and -resistant (RR) *S. frugiperda* and their F_1_ crosses (RS) on Cry1F maize leaf tissue was determined using the same method as described in the F_2_ screen (Table S9). The RR was collected from Puerto Rico in 2011 and was documented to be highly resistant to Cry1F protein and Cry1F maize plants^25,36^. The baseline survival assays showed a survivorship of 0, 1.2, and 58.6% on HX1 leaf tissue for SS-FL, RS, and RR, respectively. Thus, the expected number of survivors in the F_2_ of an family was estimated as {96 × [0 × f_SS_ + 0.012 × f_RS_ + 0.586 × f_RR_]}(Table S10**)**. Based on a χ^2^-test at α = 0.05, the two parents of a family were considered to possess zero RA (a genotype of SSSS) if no large larvae (≥3^rd^ instars) survived in the F_2_ screen, one RA (RSSS) if 1–7 large larvae survived, two RAs (RRSS or RSRS) if 8–21 large larvae survived, three RAs (RRRS) if 23–41 large larvae survived, and four 4 RAs (RRRR) if ≥42 large larvae survived. The criteria left only one undecided situation when there were 22 survivors (i.e., FL-CL-45), which resulted in a χ^2^-value of 4.37 (*P* < 0.05) for two RAs or 4.77 (*P* < 0.05) for RRRS. Because the χ^2^-value for two R alleles is smaller than the value for RRRS, the parents were considered to possess two R alleles if there were 22 survivors.
