# Supplementary material for: Cry1F Resistance in Fall Armyworm Spodoptera frugiperda: Single Gene versus Pyramided Bt Maize
Source: PLoS One. 2014 Nov 17;9(11):e112958. doi: 10.1371/journal.pone.0112958 (PMC4234506; doi:10.1371/journal.pone.0112958)
Supplement: Table S8 — Larval survivorship (%) of potential positive families of Spodoptera frugiperda on leaf tissue of Cry1F maize plants. (DOCX) [file pone.0112958.s008.docx]

**Table S8**. Larval survivorship (%) of potential positive families of *Spodoptera frugiperda* on leaf tissue of Cry1F maize plants.

| Insect | Total no. larvae | Survivorship |
| --- | --- | --- |
|  |  |  |
| SS-FL | 256 | 0.0 |
| LA-RD-24 | 256 | 33.6 |
| LA-RD-34 | 256 | 28.9 |
| LA-RD-37 | 256 | 53.1 |
| FL-13 | 128 | 25.8 |
| FL-39 | 128 | 25.8 |
| FL-37 | 128 | 21.9 |

SS-FL: Cry1F-susceptible strain collected from FL in 2011. Larval survival was checked 7 d after release of neonates on Cry1F maize leaf tissue using the same method as used in the F_2_ screen. Larval survival of three families (LA-RD-34, LA-RD-37, and FL-39) on the non-Bt maize leaf tissue ranged from 46.9% to 64.1% with an average of 56.8%. Survival on non-Bt maize leaf tissue was not assayed for the other three families.
