# Supplementary material for: Cry1F Resistance in Fall Armyworm Spodoptera frugiperda: Single Gene versus Pyramided Bt Maize
Source: PLoS One. 2014 Nov 17;9(11):e112958. doi: 10.1371/journal.pone.0112958 (PMC4234506; doi:10.1371/journal.pone.0112958)
Supplement: Table S9 — Baseline survival (mean ± SEM) of Cry1F-susceptible (SS-FL), -resistant (RR), and -heterozygous (RS) genotypes of Spodoptera frugiperda on leaf tissue of HX1 and non-Bt (NBt) maize plants. (DOCX) [file pone.0112958.s009.docx]

**Table S9**. Baseline survival (mean ± SEM) of Cry1F-susceptible (SS-FL), ‑resistant (RR), and ‑heterozygous (RS) genotypes of *Spodoptera frugiperda* on leaf tissue of HX1 and non-Bt (NBt) maize plants.

| Insect genotype | NBt-1 | HX1 |
| --- | --- | --- |
| SS-FL | 54.7 ± 4.1 b | 0.0 ± 0.0 a |
| RS | 84.4 ± 2.2 d | 1.2 ± 0.6 a |
| RR | 75.8 ± 7.1cd | 58.6 ± 5.2 bc |
| Analysis of variance | *F*_2,30_ = 7 0.66, *P* < 0.0001 for insect; *F*_1,30_ = 333.81,. *P* < 0.0001 for maize; *F*_2,30_ = 47.55, *P* < 0.0001 for interaction | |

Mean values in the table followed by the same letter were not significantly different at α = 0.05 (Tukey's HSD test).
