# Supplementary material for: Cry1F Resistance in Fall Armyworm Spodoptera frugiperda: Single Gene versus Pyramided Bt Maize
Source: PLoS One. 2014 Nov 17;9(11):e112958. doi: 10.1371/journal.pone.0112958 (PMC4234506; doi:10.1371/journal.pone.0112958)
Supplement: Table S11 — Six different parental (P1) crosses of two-parent families and the related frequencies and genetic models. (DOCX) [file pone.0112958.s011.docx]

**Table S11. S**ix different parental (P_1_) crosses of two-parent families and the related frequencies and genetic models.

| P_1_ cross | Frequency of cross | Genetic model |
| --- | --- | --- |
| RR × RR | p_RRRR_ | p^4^ |
| RS × RR and RR × RS | p_RSRR_ | 4 p^3^ (1-p) |
| SS × RR and RR × SS | p_SSRR_ | 2 p^2^ (1-p)^2^ |
| RS × RS | p_RSRS_ | 4 p^2^ (1-p)^2^ |
| SS × RS and RS × SS | p_SSRS_ | 4 p (1-p)^3^ |
| SS × SS | p_SSSS_ | (1-p)^4^ |
