# Supplementary material for: Cry1F Resistance in Fall Armyworm Spodoptera frugiperda: Single Gene versus Pyramided Bt Maize
Source: PLoS One. 2014 Nov 17;9(11):e112958. doi: 10.1371/journal.pone.0112958 (PMC4234506; doi:10.1371/journal.pone.0112958)
Supplement: Methods S1 — Supplementary methods. (DOCX) [file pone.0112958.s012.docx]

**Supplementary Methods**

**Statistical analysis for computing the expected resistance allele frequency**

To estimate the frequency of a RA in the sample, it is necessary to have a Bayesian model that produces a posterior distribution for the frequency of resistance based on the observed data and prior information about the frequency, and it is necessary to have a genetic model that connects the statistical inferences from the Bayesian model to the transmission genetics of resistance alleles.

When resistance is recessive and determined by a single allele at one locus, several variations on F_1_ and F_2_ screens can be used to estimate resistance allele frequency from the data [39, 46, 47, 48, 50]. However, when additional information about the pedigrees from the screened families can be determined (e.g., the number of resistance alleles in the parental generation (P_1_)), this information can be used to improve the estimate of the resistance allele frequency.

The Bayesian statistical model is a multinomial problem. Suppose it is possible to distinguish six different P_1_ crosses (Table S11) for an autosomal locus. This can be expanded to cover cases where all reciprocal crosses can be distinguished, and can be simplified if fewer crosses can be distinguished. Let *f*(***π***|***α***) be the prior distribution of ***π*** = (*π*_1_, … , *π_c_*), which is the vector of prior estimates of the frequency of each cross, here designated *c* = 1 to 6(corresponding to the genetic cross in Table S11), and ***α*** is the vector of hyperparameters that characterize the prior information. Let **X** be the set of observations and *L*(***π***|**X**) the likelihood of ***π*** given **X**, so that *f*(***π***|**X**,***α***) *L*(***π***|**X**) *f*(***π***|***α***) as determined by Bayes’ rule. The multinomial case is well known^51,52^ and **X** = (*n*_1_, … , *n_c_*), which is the number of observations in each of the cross types. $L(\mathbf{\pi}|\mathbf{X})\propto\prod_{i=1}^{c} \pi_{i}^{n_{i}}$, and the conjugate density function is the Dirichlet, which expressed in terms of gamma functions is$f(\mathbf{\pi}|\mathbf{\alpha})=\frac{\Gamma\left(\sum\alpha_{i}\right)}{\prod_{i} \Gamma(\alpha_{i})}\prod_{i=1}^{c} \pi_{i}^{\alpha_{i}-1}$. The Dirichlet is the multinomial analogue to the beta density function.

The genetic model relates each of the *π_i_* to an expected frequency based on the resistance allele frequency, *p*. These are calculated based on simple Mendelian inheritance of the resistance allele under Hardy-Weinberg equilibrium, and are given in Table S11. When these expected frequencies are substituted into the multinomial likelihood function, it simplifies to

$L(\mathbf{\pi}|\mathbf{X})\propto p^{\left(4n_{RRRR}+3n_{RSRR}+2n_{SSRR}+2n_{RSRS}+n_{SSRS}\right)}(1-p)^{\left(n_{RSRR}+2n_{SSRR}+2n_{RSRS}+3n_{SSRS}+4n_{SSSS}\right)}\propto L(p|\mathbf{X})$.

This is a binomial likelihood function, *L*(*p*|**X**). Similarly the Dirichlet simplifies to a beta function, which makes intuitive sense because the beta is the conjugate density function for the binomial likelihood function. If the prior distribution of *p* is designated Beta[*a*, *b*], then the posterior distribution of *p* is Beta[*a* + 4 *n_RRRR_* + 3 *n_RSRR_* + 2 *n_SSRR_* + 2 *n_RSRS_* + *n_SSRS_* ,*b* + *n_RSRR_* + 2 *n_SSRR_* + 2 *n_RSRS_* + 3 *n_SSRS_* + 4 *n_SSSS_*]. From this, all of the statistical machinery developed for the F_2_ screen can be used to compute the maximum likelihood estimator of *p* and its 95% credibility limits. When the SSRR and RSRS crosses cannot be distinguished, the posterior density simplifies to Beta[*a* + 4 *n_RRRR_* + 3 *n_RSRR_* + 2(*n_SSRR_* + *n_RSRS_*) + *n_SSRS_* ,*b* + *n_RSRR_* + 2(*n_SSRR_* + 2 *n_RSRS_*) + 3 *n_SSRS_* + 4 *n_SSSS_*]. Here we assume a uniform prior with *a* = *b* = 1.

**Supporting References**

49. Yue B, Huang F, Leonard BR, Moore S, Parker R, et al. (2009) Verifying an F_1_ screen for identification and quantification of rare *Bacillus thuringiensis* resistance alleles in field populations of sugarcane borer, *Diatraea saccharalis*. Entomol Exp App 129: 172−180.

50. Andow DA, Bentur JS (2010) Pedigreed crosses to estimate recessive virulence allele frequencies in natural populations of gall midges. Entomol Exp App 135: 18−36.

51. Lindley DV (1964) The Bayesian analysis of contingency tables. Ann Math Stat 35): 1622−1643.

52. Good I (1965) The estimation of probabilities: An essay on modern Bayesian methods. Cambridge, MA, USA. 109 p.
